# The longstanding challenge of the nanocrystallization of 1,3,5-trinitroperhydro-1,3,5-triazine (RDX)

**DOI:** 10.3762/bjnano.8.49

**Published:** 2017-02-17

**Authors:** Florent Pessina, Denis Spitzer

**Affiliations:** 1 NS3E, UMR 3208 ISL-CNRS-Unistra, Institut franco-allemand de recherches de Saint-Louis (ISL), 5 rue du Général Cassagnou, F-68301 St. Louis, France

**Keywords:** crystallization, nanoparticles, RDX, review, submicrometer, 1,3,5-trinitroperhydro-1,3,5-triazine

## Abstract

Research efforts for realizing safer and higher performance energetic materials are continuing unabated all over the globe. While the thermites – pyrotechnic compositions of an oxide and a metal – have been finely tailored thanks to progress in other sectors, organic high explosives are still stagnating. The most symptomatic example is the longstanding challenge of the nanocrystallization of 1,3,5-trinitroperhydro-1,3,5-triazine (RDX). Recent advances in crystallization processes and milling technology mark the beginning of a new area which will hopefully lead the pyroelectric industry to finally embrace nanotechnology. This work reviews the previous and current techniques used to crystallize RDX at a submicrometer scale or smaller. Several key points are highlighted then discussed, such as the smallest particle size and its morphology, and the scale-up capacity and the versatility of the process.

## Review

### Introduction

While nanotechnology has spread to nearly all other sub-fields of material science, the pyrotechnic community has struggled to produce very fine particles of organic explosives. Beside the technical considerations, scientists are also striving to convince the quite conservative military industry of the added value of energetic nanomaterials. While the interest in nanoparticles has been recently highlighted, companies continue with process optimization [1] and observe the technological breakthroughs of the last decades with caution. As a consequence, the innovation has been mainly focused on the synthesis and prediction of new molecules such as 1,3,3-trinitroazetidine (TNAZ), 2,4,6,8,10,12-hexanitro-2,4,6,8,10,12-hexaazaisowurtzitane (CL-20), octanitrocubane (ONC), 1,1-diamino-2,2-dinitroethene (FOX7), ammonium dinitramide (ADN), and 5-nitro-1,2,4-triazol-3-one (NTO). These new materials aim to achieve higher density, to increase the processability and to attain insensitive munition (IM) characteristics; however, IM properties actually rely on the whole physics-chemistry of the system. Therefore, the development of powders with controlled particle size and morphology and well-defined surface chemistry is largely unexplored for energetic materials at the sub-micrometer scale and smaller. The criteria that are advantageous for new energetic materials include the following:

- high decomposition temperature
- low sensitivity
- no phase transitions under compression or depression
- no autocatalytic decomposition
- no voids from solvents or gas
- mechanical behavior independent from temperature
- good availability/cost ratio
- easy processing

The compression of gaseous inclusions, cavities and voids, deformation, frictional heating, intercrystalline shearing and spark discharge (electrostatic discharge (ESD)) are initiation processes which can cause areas of an energetic material to heat up to several hundred Kelvin. These areas are called hot spots and are deflagration or detonation origins if they reach a critical temperature. Tarver [2] calculated the critical temperature for octahydro-1,3,5,7-tetranitro-1,3,5,7-tetrazocine (HMX) of different sized hot spots. For a 2 μm diameter hot spot he calculated a critical temperature of 985 K, whereas the critical temperature for a 0.2 μm hot spot already rises to 1162 K.

Risse [3] measured a noticeable desensitization towards initiation by friction and electrostatic discharge for nanostructured 1,3,5-trinitroperhydro-1,3,5-triazine (RDX) crystallized by spray flash evaporation (SFE), compared to the raw material (Table 1). The noticeably lower sensitivity towards friction can be based on the self-lubricating effect, as small particles will tend to occupy small interstices instead of breaking. Sensitivity measurements were also performed on hexolite, which showed a clear desensitization of the nanostructured explosive (Table 2).

**Table 1:** Sensitivity towards impact, friction and ESD of micrometer-sized and nanostructured RDX (n-RDX) [3].

RDX	Impact	Friction	ESD
	[J]	[N]	[mJ]

M5 (raw material)	*>*3.52	160	120
nanostructured RDX	*>*3.52	*>*360	270

**Table 2:** Comparison of the sensitivity levels of micrometer-sized hexolite with those of a nanometer-sized hexolite (n-hexolite) [3].

Hexolite	Impact	Friction	ESD
	[J]	[N]	[mJ]

micrometer	6	54	353.6
nanometer	25.06	72	436.6

Using a sonocrystallization process, Bayat and Zeynali [4] succeeded in the preparation of n-2,4,6,8,10,12-hexanitro-2,4,6,8,10,12-hexaazaisowurtzitane (n-CL-20), which was less sensitive towards friction, impact and electrostatic discharge (Table 3).

**Table 3:** Comparison of the sensitivity levels of micrometer and nanometer-sized CL-20 [4].

Particle size	Impact	Friction	ESD
[μL]	[cm]	[kg]	[J]

15	25	6.4	45
0.095	55	no reaction	60

**Figure 1:**
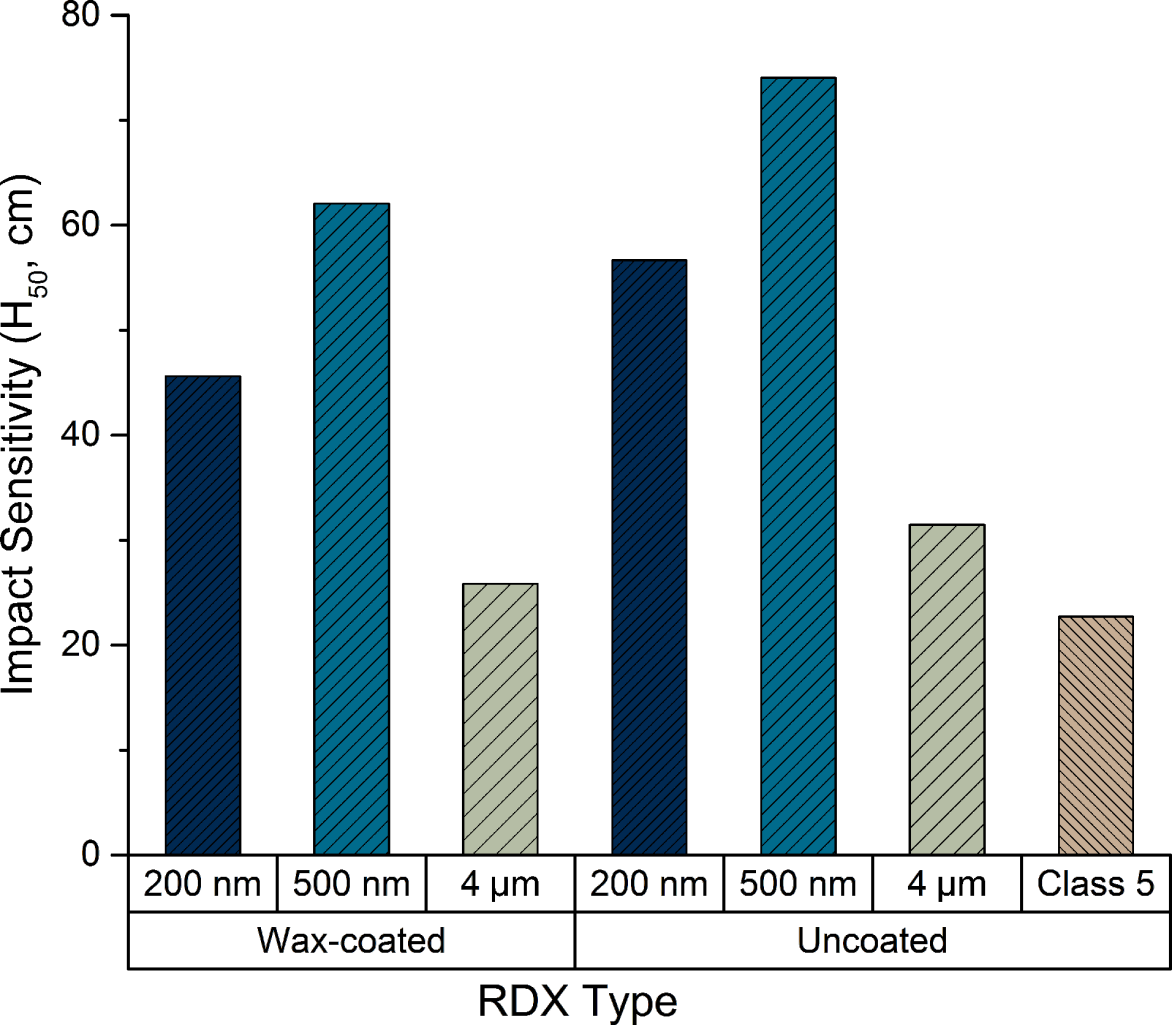
Impact sensitivity of RDX as a function of RDX type and particle diameter, adapted from [6].

Klapötke has often experienced the influence of particle size on ESD, when stating for example ”the finer the powder of a particular (note from authors: RDX) sample is, the higher the ESD sensitivity values are” [5]. However, this trend is not always observed. Crystallized from rapid expansion of supercritical solutions (RESS), several nanometer-scale RDX (n-RDX) lots have been tested by Stepanov et al. [6]; while both 500 nm and 200 nm diameter RDX are less sensitive toward impact than milled 4 μm RDX, the 200 nm diameter lot is substantially more sensitive than the 500 nm one. As it can be seen in Figure 1, the minimum sensitivity to impact is confirmed when coating the powders with a binder; however, that confirmation might reveal that the trend is more due to the intrinsic bulk properties of the particles instead of their surface. Klaumünzer et al. [7] investigated the sensitization of n-RDX against impact and strongly reaffirmed that the generalization of a direct link between smaller particle and lower impact sensitivity – as seen many times in the literature – should be more forcefully addressed.

Beside safety, other improvements can emerge from materials prepared on the nanoscale. The critical diameter, tunable detonation velocity, delay before the detonation steady state, etc. may be improved by the drastic grain size reduction. Energetic nanocomposites are also promising materials improved by a more intimate mixing. Liu et al. [8] showed that detonation velocities of PBX composition from their milled n-RDX and n-HMX materials are slightly better while being significantly safer. The burning rate of nitrocellulose was also improved by Zhang and Weeks [9] due to the formation of sub-micrometer spheres. Recently, Comet et al. [10] proved that energetic nanocomposites can easily replace the hazardous primary explosives to initiate a secondary explosive: 500 nm diameter n-RDX from SFE was mixed with a nanoscale thermite to initiate the detonation of PETN. The flame propagation velocity (FPV) of composites made of n-WO_3_/n-Al/n-RDX can be tuned from 0.2 to 3.5 km/s through their explosive content. Under the same conditions, n-WO_3_/n-Al with μ-RDX exhibit unstable regimes from 187 to 733 m/s, whereas the n-WO_3_/n-Al/n-RDX composite deflagrates at a constant velocity of 2529 m/s. Those and other unpublished results from our laboratory confirm the drastic reduction of the critical diameter with the decrease of particle size.

1,3,5-Trinitroperhydro-1,3,5-triazine is also found under the following descriptors: cyclo-1,3,5-trimethylene-2,4,6-trinitramine, 1,3,5-trinitrohexahydro-s-triazine, cyclotrimethylenetrinitramine, hexahydro-1,3,5-trinitro-s-triazine, trimethylenetrinitramine, T4, cyclonite, hexogene and RDX. The most common and widely accepted one is the acronym RDX and is the one used in this present work. With nitro groups and a triazine cycle, it is chemically similar to many organic explosives and thus a good representative sample. Additionally, although it was created in the 1930s, RDX is still widely used for civilian and military applications, reinforcing its interest in industrial and scientific studies. Therefore, RDX has been used over the years as a reference for crystallization experiments, aiming to reduce the size of energetic organic crystals below a micrometer. Many crystallization processes inspired from other fields of chemistry, such as polymer crystallization, have been applied but only a few resulted in a significant size reduction.

The present review aims to fairly depict all the crystallization processes used on the same material, RDX, and the limitations encountered. The smallest particle size is of interest and will be offset against the maturity of the technology. The interest for a process can be also expressed according to its versatility and its ability to process continuously as per industrial standards. Such key points are further discussed in the conclusion. The classification suggested here is based on the final state of the particle formation. If a significant additional step is required to dry the particles, which is often because the crystallization occurs in a liquid that does not simultaneously evaporate or is not in a supercritical state, the technique is classified as a wet production method. There is only one notable exception, the aerosol solvent extraction system (ASES), sorted among the supercritical processes for a better understanding. Melting and milling processes for producing sub-micrometer energetic materials require one or more additional liquids, therefore these techniques are classified as wet methods.

### Wet production methods

#### Crystallization from solution

Crystallization from solution has been studied but also used at the industrial scale for centuries. From solution, hydraulic processes can be easily monitored, controlled, and continuously operated. Therefore, substantial efforts have been made to scale the crystallization of energetic particles with existing technologies.

Depending on the creation of supersaturation, distinctions are made between cooling, evaporation, vacuum cooling, drowning-out and reaction crystallization. The study of the solubility of the compound is the key to determine which crystallization process can be used. For instance, if the solubility is not very temperature dependent, evaporation will be more effective than cooling. To our knowledge, no consistent study of the behavior of the solubility of RDX has been made. Fedoroff and Sheffield [11] indicate that the RDX solubility in acetone is reduced four times by cooling from 60 to 0 °C. Pant et al. [12] used all standard techniques available to recrystallize RDX into sub-micrometer crystals in a beaker. The smallest size was obtained when the antisolvent was added to a cooled, highly supersaturated solution, while applying ultrasonication and stirring. Achieving a high level of supersaturation results in a higher nucleation rate, but aggregation increases rapidly. For this solvent/compound system, they produced smaller particles and reduced the agglomeration by sonication and the particles were finally obtained by drying. This method may be suitable for industry, however, the minimum size obtained was only 850 nm under tough conditions, with a yield of 60%, resulting in rod-shaped crystals. Such morphology must be avoided due the well-established sensitization for materials with a high length-to-width ratio [13–15]. Increasing roughness due to surface defects also sensitizes the energetic materials.

Kumar et al. [16] succeeded in producing finer RDX particles by quickly injecting a very small volume (100 μL) of RDX dissolved in acetone into ultrapure water. The smallest mean particle size was 38 nm as determined by scanning electron microscopy (SEM) for the highest temperature (70 °C) and lowest concentration of RDX in acetone (5 mM). It is worth mentioning that dynamic light scattering (DLS) measurements were found to be not reliable when compared to SEM analysis, which can be explained by the lack of surfactant to stabilize the colloid. This technique was also applied on HMX [17] with a particle size around 30 nm and the same conclusions were drawn.

Bayat et al. [18], through an optimization of the microemulsion process, crystallized 80 nm plate-like β-CL-20 particles. The severe agglomeration and plate-like morphology could be due to the freeze drying and washing of the microemulsion. Gao et al. [19] recrystallized 1,1-diamino-2,2-dinitroethene (FOX7) in ethyl alcohol down to the sub-micrometer range. SEM pictures show an irregular plate-like morphology and therefore highlight the inconsistency of the unique mean particle size of 340 nm claimed. The particles also exhibit a certain degree of agglomeration which can be explained again by the pool of surfactant and the air-drying method used.

Luo et al. [20] reached an impressive mean diameter of 30 nm of RDX. They used an unusual technique where RDX is dispersed in bacterial cellulose. The smallest particle size was obtained with a 71% RDX/gelatine mixture. However, increasing the content of RDX leads to an increase in the particle size and the maximum RDX loading tested was 91% resulting in a mean particle diameter of 50 nm. The sensitivity of this composite towards impact and friction was reduced by two times, therefore questioning the reactivity. Nevertheless, further efforts were made to replace the bacterial cellulose with an energetic matrix.

Crystallization in solution allows the formation of large crystals by growth, thus allowing more parametric studies regarding the influence of solvents. For instance, one work [21] has studied the importance of temperature and supersaturation for the crystallization for HMX in γ-butyrolactone, revealing that low temperature and highly supersaturated solutions tend to increase the defects in HMX crystals.

#### Solvent substitution using reverse micelles

Dabin et al. [22] have developed an ingenious method to prepare nanometer-scale RDX using a simple technique. The crystallization is triggered by a solvent substitution, and the nanometer scale material is obtained by restricting the reactor volume using reverse micelles. NaAOT (sodium 1,4-bis(2-ethylhexoxy)-1,4-dioxobutane-2-sulfonate) with isooctane was used to form reverse micelles. Then RDX in dimethylformamide (DMF) is added to one solution containing these micelles, and water to another solution of micelles. Both are finally mixed together to form the n-RDX with a diameter of 70 to 100 nm. However, the nanoparticles produced from this elegant solution exhibit an undesired rod-like morphology.

#### Sol–gel

Energetic materials processed by the sol–gel method are desensitized by being embedded in a matrix, usually a silica one. Developed by Gash et al. [23] and Tillotson et al. [24–25], the silica explosive gels are prepared by dissolving the energetic compound, the silica precursor and a catalyst in a co-solvent. After the gelification, an antisolvent of the explosive is injected to replace the solvent in the pores and precipitate the explosives in the silica matrix. By drying with heating or at low pressure, a xerogel with higher density is obtained. If supercritical CO_2_ is used to extract the solvent, an aerogel with low density is formed. Therefore, the nanostructured nature of the explosive comes from the porous matrix: cavities of mesoporous gels are 2 to 50 nm large and less than 2 nm for microporous gels. Macroporous materials have pore diameters greater than 50 nm [26].

An RDX/resorcinol formaldehyde (RF) nanocomposite has been synthesized [27] where 38 nm RDX crystallized in an RF aerogel matrix with a surface area of 551.5 m^2^/g (measurement taken without RDX). Wuillaume et al. [28] trapped ammonium perchlorate (AP) and RDX in a mesoporous low-density energetic organogel. During the impact test, a negligible decrease of sensitivity was measured: 75 wt % RDX nanogels and macrogels had the same sensitivity and the 90 wt % nanogels are even more sensitive than their macroscopic counterparts. When compared to pure RDX, the 90 wt % nanogels are not desensitized. However, small scale gap tests (SSGTs) preformed on pressed gels (95% TMD) revealed an improvement of the sensitivity for the 90 wt % RDX nanoformulation. The nanogel exhibits an uncommon microstructure of sheets, with micrometer-sized particles potentially formed by primary nanoparticles. The lack of desensitization in the loose powder may be explained by the sensitization by the sheet-like shape counteracted by the presence of the gel coating each nanoparticle.

Li et al. [29] used a better energetic matrix, GAP, with a maximum of 40 wt % RDX. They noticed a lower sensitivity than the physical mix. However, the claimed nanometer diameter is only deduced from the porosity without RDX and from X-ray diffraction (XRD) patterns which only give the mean coherence length. They also created NC-RDX-AP nanocomposites using a technique similar to sol–gel [30]. The matrix is the NC itself solidified by micrometer-sized AP crystals and cross-linked with toluene diisocyanate (TDI) and dibutyltin dilaurate (DBTDL), whereas RDX is dissolved in acetone inside that template. The gel and the crystallization of RDX is triggered by supercritical CO_2_ drying. Even if the sensitivity and the density were not improved, the increase of the heat of explosion measured and the originality of the approach make the formation of a nanocomposite based entirely on energetic materials through chemical binding promising.

#### Melting

Many high energetic materials degrade very close to their melting point. Therefore, only a few such as 2,4,6-trinitrotoluene (TNT) or TNB can be used in the molten state since the melting temperature is at least 100 °C away from the exothermic decomposition. The melt–cast process of TNT-based compositions has been used for shaping charges or loading them into ammunitions since World War I. Crystallization from an emulsified molten explosive is an innovative technique used by Anniyappan et al. [31]. 2,4,6-triazido-1,3,5-triazine (or cyanuric triazide, CTA) has been processed at 95 °C to crystallize as micrometer-sized agglomerates. CTA is a promising primary explosive compliant with the new REACH legislation [32] forbidding the use of heavy-metal-based materials. Further investigation with surfactants might lead to smaller particles by counteracting the high viscosity of molten droplets.

#### Milling

Redner et al. [33] developed a batch wet-milling process, producing sub-micrometer RDX. A mixture of water, isobutanol, a dispersant and RDX was filled into an unspecified mill, resulting in a minimum mean particle size of 310 nm and a crystallite size of about 65 nm. Several milling issues were mentioned such as the yield of about 25% and the importance of the residence time and the drying step.

Liu et al. [34–35] studied the effect of drying n-RDX and HMX samples under various conditions. They first obtained n-RDX in solution from a mixture of water, ethanol, isopropanol and RDX. The suspension was put in a bidirectional rotation mill for 6 h. Just as Rednere experienced, the drying process is a critical step to obtain a nanogranular powder. They dried the RDX under different conditions: freeze drying and supercritical drying led to quite impressive results, with median diameters of 160 nm and 200 nm, respectively from a solution containing an average particle size of 64 nm. After RDX and HMX, CL-20 was successfully processed the same way resulting in a median diameter of 180 nm, as determined by SEM [8]. For the three compounds, the nanopowders were found to be less sensitize than their micrometer-sized counterparts.

Spray drying is a less energy intensive RDX drying method studied by Patel et al. [36]. RDX and CL-20 were bead milled from water with the addition of isobutanol and poly(vinyl alcohol) (PVOH) to stabilize the colloid by dispersion and coating. Then, an unknown polymeric binder was added just before drying the slurry by spray drying. Mean particle diameters down to 400 and 200 nm have been measured by DLS for RDX- and CL-20-based composites, respectively, after milling. However, no particle size distribution (PSD) curve was provided nor was the dispersion of the results indicated. It has been noticed that for 200 nm particles of CL-20 the α phase is obtained. From the same research group, nanoscale CL-20:HMX has been prepared by bead milling an aqueous suspension of ε-CL-20 and β-HMX in a 2:1 stoichiometric ratio [37]. The progressive conversion of raw materials into the co-crystal is achieved after one hour, resulting in a particle diameter of less than 200 nm. However, not much attention was paid to the drying effect of large-scale batches. SEM and XRD measurements were performed on a drop-dried material at room temperature, and it is likely that the drying of several grams of such molecular crystal will behave differently. Furthermore, the accuracy of the XRD technique does not allow one to conclude that a complete conversion into the co-crystal has occurred, but rather indicates that the percentage of ε-CL-20 and β-HMX is lower by approximately 10%. The full quantification by Rietveld or full pattern matching methods would have been useful to follow the conversion with time.

### Dry production methods

#### Physical vapor deposition (PVD)

In 2002, Frolov and Pivkina first reported on a vacuum condensation process for high energetic materials [38–40]. The vacuum deposition of ammonium nitrate (AN), RDX and a composite AN–RDX was performed on a cooled quartz substrate. The mean particle diameter was directly measured from atomic force microscopy (AFM): a diameter of 50 nm was obtained for the three materials, even after processing the nanopowder (removal from the quartz substrate and pressing into tablets).

Mil’chenko et al. [41] delved further in the physical vapor deposition (PVD) process with the deposition of 2,4,6-triamino-1,3,5-trinitrobenzene (TATB), HMX, RDX, PETN and BTF as thin layers on several substrates such as plexiglas and copper while changing operative parameters. The critical thickness of the detonating layer is an order of magnitude lower; the sensitivity toward impact and friction is barely mentioned as being similar to the raw materials but the sensitivity to laser excitation has been substantially increased.

Therefore, the PVD technique is highly suitable for pyrotechnic integrated circuits or micro-electromechanical systems [42–43], whereas mass production of loose powder is not economically viable.

#### Electrospray

Radacsi et al. [44] crystallized sub-micrometer RDX using an electrospray technique. A solution of RDX/acetone is sprayed through a nozzle that is electrically charged to a grounded plate. This charges the droplet surface, increasing the surface energy and thus triggering the fission into smaller droplets. This Coulomb fission phenomenon and the evaporation of the solvent leads to crystallization and the deposition of nonagglomerated particles. By adjusting the nozzle parameters and the potential difference various morphologies of RDX particles resulted. For instance, micrometer-sized hollow spheres of agglomerated RDX were produced. The minimum mean size was 400 nm. This sub-micrometer RDX sample exhibited a clear insensitivity towards friction, but with the same impact sensitivity as conventional micrometer-sized RDX (Table 4).

**Table 4:** Comparison of the sensitivity levels of conventional and 400 nm diameter RDX.

RDX	Impact	Friction
	[J]	[N]

conventional	7.5	120
sub-micrometer	10	*>*360

Reus et al. [45] then processed bicomponent systems: proteins and RDX/TNT. It is mentioned that the XRD patterns of the final products differ from that of the raw material, which seems to indicate either a strong degradation or cocrystallization. Infrared spectroscopy definitely demonstrated a critical partial decomposition of both RDX and TNT due to the electrospray technique and the same phenomenon has likely happened for Radacsi et al., too. Whatever was really obtained, Reus crystallized very small particles, estimated by us to be less than or approximately 100 nm for any initial ratio of TNT/RDX. AFM measurements could have provided much more information about the size and shape of such nanoparticles, especially since they are well dispersed on a substrate. The sensitivity tests have been performed on those degraded materials, which were found to be as insensitive as TNT.

The electrospray technique can be used to create a fine spray of micrometer-sized charged droplets repelling each other, which is ideal for crystallization. The high voltage needed is a major handicap for processing sensitive powders such as energetic materials containing nitro groups.

#### Plasma

During his Ph.D. project [46], Radacsi used an innovative and advanced technique to crystallize sub-micrometer RDX: a collison nebulizer that aerosolizes an RDX/acetone solution to a surface dielectric barrier discharge (SDBD) plate where a cold plasma disrupts the droplet by the Coulomb fission. Like the electrospray technique, each droplet should crystallize into a unique single crystal. The minimum mean diameter obtained was 500 nm, with a diameter distribution from 200 to 900 nm, and the particles had a prismatic or spherical shape. Again, like the sub-micrometer powder obtained from the electrospray technique, the 500 nm RDX was desensitized to friction but not to impact (Table 5).

**Table 5:** Comparison of the sensitivity levels of conventional and 500 nm diameter RDX [46].

RDX	Impact	Friction
	[J]	[N]

conventional	5	144
sub-micrometer	5	*>*360

#### Supercritical/gas antisolvent precipitation

Supercritical antisolvent (SAS) precipitation uses the same principle as liquid crystallization, substituting the liquid antisolvent by a supercritical fluid. The very high diffusivity of supercritical fluids leads to a rapid supersaturation and thus to a sudden precipitation. Various approaches are used in SAS. One is the supercritical antisolvent (GAS) precipitation, where the liquid solution is first loaded into the vessel before the addition of the supercritical antisolvent. For RDX, CO_2_ is an appropriate supercritical antisolvent. Gallagher et al. [47] first investigated the use of the GAS process for RDX in 1992. Supercritical CO_2_ injected into an RDX/cyclohexanone solution at various injection times, injection quantities and temperatures. In this first use of GAS for RDX, various particle sizes and morphologies were obtained, but none under the micrometer scale. Since then, several process derived from the GAS process (which could be referred as SAS subprocesses) have been used to form particles on the sub-micrometer and nanoscale for energetic materials. However, since 1992, no GAS/SAS process has been reported to produce energetic materials with a diameter less than a micrometer [48–51], except for 5-nitro-1,2,4-triazol-3-one (NTO) by Lim et al. [52–53].

#### Aerosol solvent extraction system (ASES) process

The aerosol solvent extraction system (ASES) process involves precipitation through spraying the solution through an atomization nozzle into supercritical CO_2_. Lee et al. [51] used GAS and ASES systems to crystallize β-HMX. However, undesirables shapes (needle-like, irregular and aggregated) were produced by ASES at all operating conditions, whereas GAS led to regular shapes with the desired β-phase. Dou et al. [54] sprayed RDX dissolved in DMF to obtain micrometer-sized particles of high polydispersity. However, sub-micrometer-sized polymers and biopolymers produced by ASES have been reported since the 1990s by Reverchon [55] and Dixon et al. [56]. Nevertheless, that technique can still be used on NC-based composites due to its polymer-like behavior.

#### Solution-enhanced dispersion by supercritical fluid (SEDS)

The solution-enhanced dispersion by supercritical fluid (SEDS) process was developed and patented by the Bradfort University to achieve a smaller droplet size compared to the previously described SAS methods. In the SEDS process, a solution with the solvated compound is sprayed into a supercritical antisolvent gas (CO_2_ for RDX) through a nozzle with two coaxial passages. This technique can be seen as a specific implementation of the ASES process, where CO_2_ is introduced through the nozzle simultaneously with the solution. Shang and Zhang [57] produced spherical RDX particles with a mean particle diameter of 770 nm using SEDS, which finally resulted in the reduction of the particle size under to less than a micrometer.

#### Particles from gas-saturated solutions (PGSS)

Two patents ([58–59]) first described a procedure that today is called particles from gas-saturated solutions (PGSS). The PGSS technique consists of dissolving a compressed gas into a solution of the substrate in a solvent, then passing it through a nozzle. The sudden decompression leads to crystallization and thus to the formation of solid particles. Although this method is widely used on a large scale with a wide range of products (from inorganic powder to pharmaceutical compounds [60]), nothing has been reported [49] concerning energetic materials processed by PGSS.

#### Rapid expansion of supercritical solutions (RESS)

The rapid expansion of supercritical solutions (RESS) concept was first described by Hannay and Hogart more than a century ago [61] but studied by Krukonis [62] and the Battelle Institute research team [63–64] in more detail. The RESS process consists of spraying a supercritical (sc) fluid containing the substrate through a nozzle in a low pressure chamber (0–60 bar). The sudden pressure decrease leads to rapid nucleation where small particles (from micrometer- to nanometer-sized) are finally collected. The use of a supercritical fluid like CO_2_ allows the direct production of a dry, pure powder. Teipel et al. [48,65] first reported the use of RESS for energetic materials: 10 μm diameter TNT particles were crystallized in those preliminary experiments. In this work, parameters which strongly influence the crystallization in a RESS apparatus were discussed: pressure, temperature, geometry of the nozzle and mass flow. Stepanov, a member of the research group of Krasnoperov, succeeded in the fine tuning of the RESS process to prepare dried n-RDX [66–69]. The formed n-RDX particles had a mean particle diameter ranging from 110 to 220 nm and an irregular spherical morphology. He performed a scale-up of the RESS process in order to increase the production capacity of RDX to 6 g/h but with a CO_2_ consumption of 35 kg/h. Through RESS, a slight sensitization to impact and shock stimuli of the 200 nm RDX occurred compared to 500 nm RDX [6].

CL-20 has also been processed by RESS from trifluoromethane (CHF_3_) [53]. Supercritical CHF_3_ has similar thermodynamic properties and is a better solvent of CL-20 than scCO_2_. Only micrometer-sized particles could be produced and no article reporting the results could be found. Changing the solvent is an area of research followed by Lee et al. [70] using compressed liquid dimethyl ether (DME) for RDX. The parametric study points out the role of inlet pressure and temperature and the nozzle diameter. The increase of any of those three parameters increases the particle size. Therefore, the two minimal mean particle sizes of 370 and 360 nm were obtained for the lowest mass flow rate of 0.37 and 0.85 g/s of DME.

#### Rapid expansion of supercritical solutions into an aqueous solution (RESS-AS) (or RESOLV)

After the success of the RESS process, Essel et al. developed a new method based on that technique called RESS-AS, which as first reported in 2010 [71]. RESS-AS uses the versatility of the RESS process, while spraying into an aqueous solution containing a dispersant and/or growth inhibitor [72]. They reported [73] the synthesis of 30 nm RDX in a pH 7-stabilized solution. For such sizes and even at that pH, agglomeration and Ostwald ripening occur. Therefore, to avoid the degradation of the nanoparticles, a polymer coating (PEI or poly(vinylpyrrolidone) (PVP)) is necessary. It should be mentioned that no subsequent sensitivity tests have been reported, provoking the question about whether a nanopowder could have be obtained from those colloidal suspensions.

#### Laser ablation

For the first time, Gottfried et al. [74] successfully produced RDX nanoparticles using laser ablation. A near-infrared, nanosecond pulsed laser was focused on military-grade RDX pellets. The scanning mobility particle sizer (SMPS) and SEM analyses showed a particle size distribution around 64 nm for a 200 mJ pulse and a 75 mJ pulse. No further analysis has been reported, such as trace decomposition, crystalline quality, apparent density, sensitivity, etc.

#### Ultrasonic spray pyrolysis

Since the nineties, spray crystallization and synthesis has been performed using several atomizers, and among them piezoelectric transducers [75–76]. As a spray technique, the goal is to produce one particle per droplet, but here the crystallization is controlled by the drying step, an oven just after the atomization. Spitzer et al. [77–78] and Kim et al. [79] both developed an apparatus to produce dried sub-micrometer RDX from an ultrasonic transducer. After the droplet generation, the solvent is evaporated by thermal gradient applied on the flux pulled by a pump. Highly agglomerated particles 200–500 nm were produced. Gao et al. [19,80] used the same experimental setup – while the previous works of Spitzer et al. and Kim et al. are never cited – with the exception of the furnace having here a gradient of temperature in order to produce 78 nm FOX7 particles and sub-micrometer-sized CL-20:HMX cocrystals. The claimed large-scale synthesis has not been fairly investigated. For instance increasing the number of piezoelectric transducers and scaling up the furnace would require excessive amounts of electrical power, thus making the cost-effectiveness of this method doubtful.

#### Spray drying

The development of spray drying [81–82] has been expanding over the years and has recently become a suitable commercial solution at both R&D and industry scales to produce dried particles from micrometers to nanometers. The pyrotechnic community quickly discerned the advantages of this simple technique to process energetic compounds as pure and composite materials.

The process sprays a solution containing a dissolved compound or particles in suspension into a hot gaseous stream (air or nitrogen) thus crystallizing into particles and/or drying the granules. van der Heijden et al. [83] showed that spray drying is able to crystallize finer RDX particles (”from 400 nm and larger”) than their technique of precipitation into antisolvent (1 to 10 μm). Qiu et al. studied the crystallization of energetic compounds using spray drying with ultrasonic [84] or pneumatic [85] nozzles or with both type of nozzles [86]. All their experiments were done with the addition of poly(vinyl acetate) (PVAc) and resulted in micrometer-sized or sub-micrometer hollow spheres made of primary nanoparticles; the smallest of which were estimated at 20 nm for RDX/PVAc made from a pneumatic nozzle with a mean droplet size of around 7 μm. The versatility of the process allows the production of energetic composites (coating of TATB on micrometer-sized HMX, RDX or CL-20 by Ma et al. [87]), energetic/elastomer composites (micrometer-sized CL-20/EPDM by Ji et al. [88], micrometer-sized spheres of agglomerated HMX/Viton by Shi et al. [89]), and even co-crystals (micrometer-sized spheres of agglomerated HMX/TNT by Li et al. [90]).

### Spray flash evaporation (SFE)

Risse and Spitzer developed an innovative process after experiencing the limitations of the ultrasonic spray pyrolysis method: beyond the inherent risk of using a high voltage electrostatic precipitator for energetic powders, the rate of evaporation of droplets was too low to avoid agglomeration and to crystallize sub-micrometer particles. Risse et al. [3,91] used the flash-evaporation behavior of droplets to dramatically reduce the lifetime and the size of droplets. The compound is dissolved in a volatile solvent and that solution is heated just before being sprayed into vacuum, where the crystallization is triggered by the sudden temperature depression and the solvent evaporation. Due to the recent advances in that technique and its versatility, the spray flash evaporation (SFE) process deserves the following review of the corresponding literature.

#### Theoretical insights on the SFE technology

Flash evaporation is the physical phenomenon occurring when the boiling point of a liquid is lower than its actual temperature due to a sudden drop of pressure and/or a quick increase of temperature. The excess heat is instantly converted into latent heat of vaporization, cooling both liquid and vapor down to the saturation temperature. Multistage flash (MSF) evaporators of static water have been used since the middle of the 20th century [92–94] with yield of around 100 m^3^ per day, receiving interest mainly from the U.S. West Coast [95] and Japan (national research program ”seawater desalting and by product recovery” launched in 1969, [96]). Current applications are extended from solution concentration such as in wine industry [97] to heat dissipation of electronic chips and laser devices [98].

Brown and York [99] found a critical temperature above which the liquid jet burst by rapid bubbling. They injected water up to 13 bar through simple single-hole nozzles with a minimal diameter of 500 μm into ambient pressure. The linear mean droplet size was found to follow a linear variation of temperature. Then, in 1981, Miyatake et al. were pioneers in the field of flash evaporation and published the first known articles about spray flash evaporation with superheating [100–101], after studying flash evaporation from water pool [102]. Many technical limitations restricted their studies for current issues: only straight-lined liquid jets were studied with basic optical techniques where the smallest drops and bubbles could not be indexed. However, Miyatake et al. [103] interestingly used electrolysis to generate more bubbles into a flashing water jet. Nowadays, not many laboratories still investigate flashing liquid jets. Guenther and Wirth [104] characterized flashing liquid jets with modern techniques and noticed the formation of bubbles inside a glass nozzle for high superheating. They also demonstrated that a simple acoustic measurement can be used to monitor the atomization of superheated liquids. The current application of flashing liquid jet is the improvement of MSF desalination processes of sea water [105–106], where a much higher evaporation rate is obtained in contrast to static flash evaporation where the rate is surface dependent.

**Figure 2:**
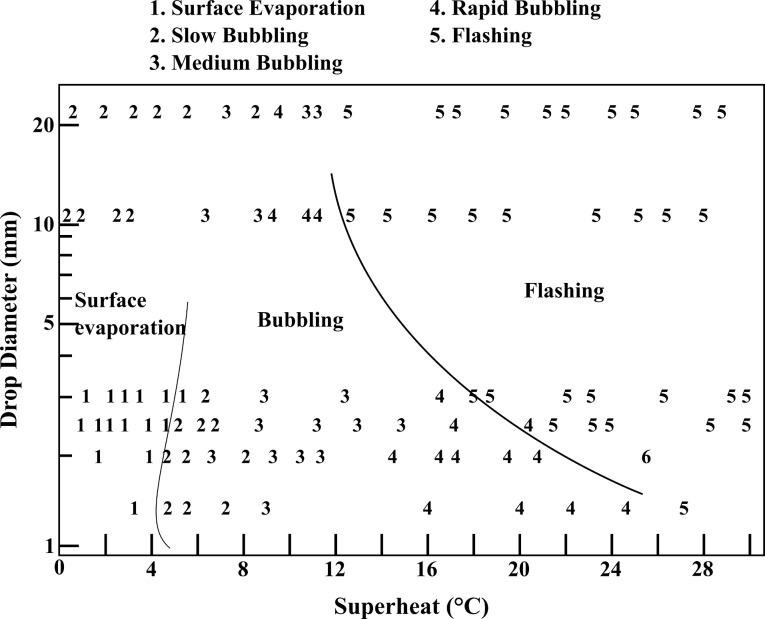
Empirical diagram of the evaporation of a water drop, adapted from [107]

We mentioned applicative publications for now, but specific studies on the flashing phenomenon of droplets are rare. Owen and Jalil [107] investigated that specific form of evaporation on isolated drops. A superheating of 0 to 5 °C triggers only surface evaporation, then boiling occurs at higher superheating. Flashing is triggered for superheating from 18 to 24 °C for a drop of 1–3 mm and larger drops flash more readily as illustrated in Figure 2. Since flash evaporation is closely related to cooling, many theoretical approaches start with a simplified model without superheating: Shin et al. [108] and Satoh et al. [109] thoroughly described the evaporation behavior of a water droplet in an abruptly evacuated atmosphere leading to its solidification. Sobac et al. [110] developed a comprehensive model of the evaporation of a liquid spherical drop that is not applicable to extremely small droplets as in flashing spray.

Interesting studies close to the current SFE process came from Gebauer et al. [111–114]. In their system, a pressurized, superheated liquid is atomized through an hollow cone nozzle into a low pressure chamber and micrometer-sized particles are recovered. However, only a partial evaporation occurs and leads to further crystal growth during the flight time and is deposited in the sump collected in the bottom of the crystallizer.

#### Comprehensive description of SFE

**Figure 3:**
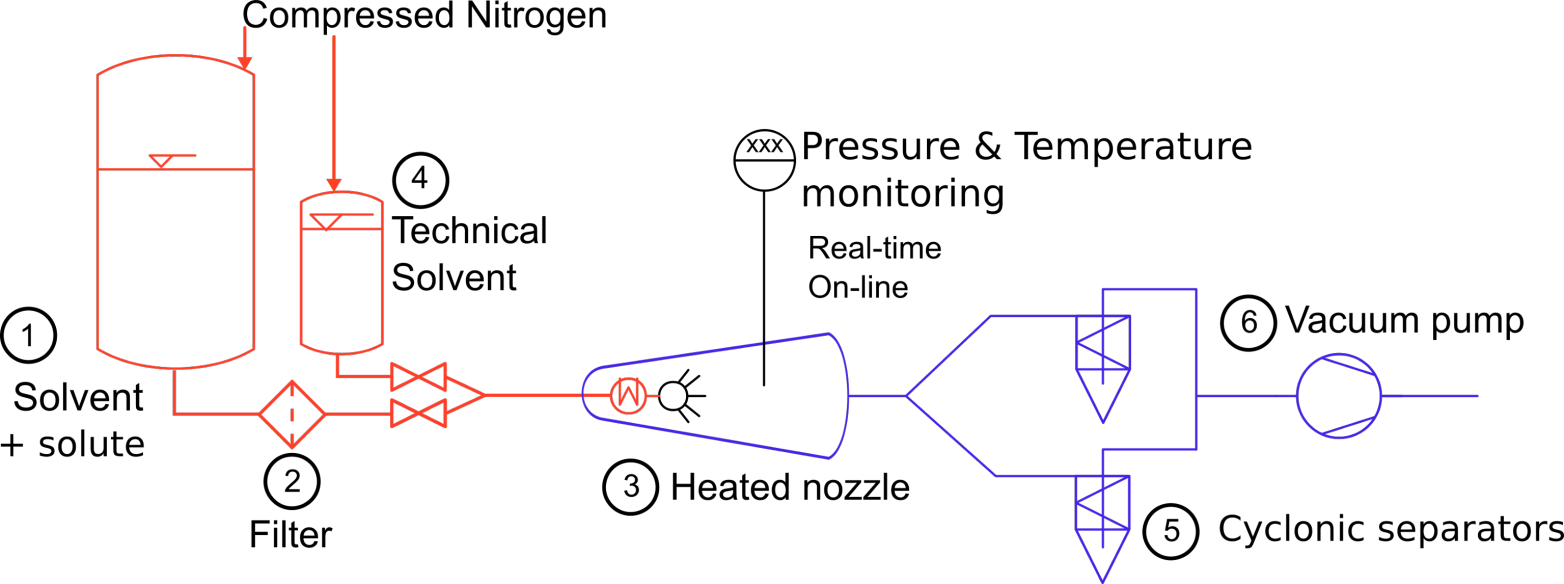
SFE installation as patented and used in this present work

**Figure 4:**
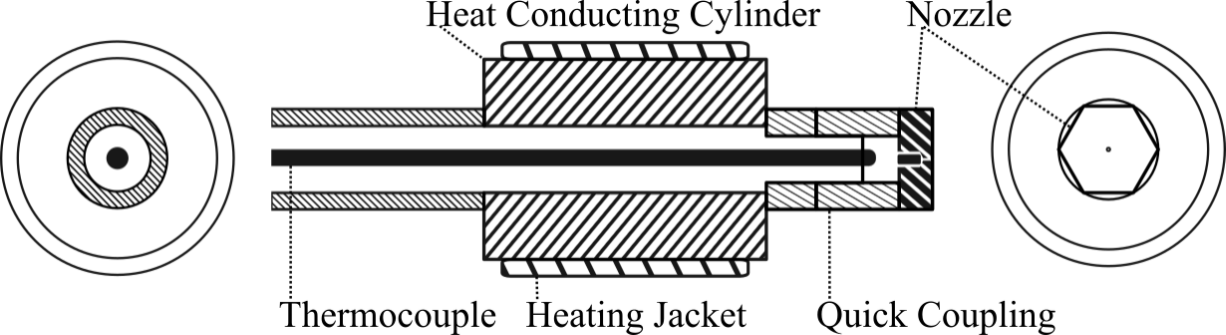
Schematic cross-sections of the nozzle and its heating system; from left to right, rear view, longitudinal cross-section and front view.

Figure 3 describes a standard SFE apparatus, where two zones can be distinguished: a high pressure one is in red and the low pressure one in blue. One storage tank (**4**) is used for technical operation such as preheating, postcooling and cleaning and is filled with technical grade solvent. The compounds of interest are dissolved in solvent in an other tank (**1**). Both tanks are pressurized with a dry carrier gas up the pre-expansion pressure. the fluid is brought inside the atomization chamber at this pressure using standard industrial hydraulic tubes; there, the liquid is superheated within a jacket around a metallic heat conductor. A regulation is made within a thermocouple (type K, diameter 1.5 mm) plugged to a proportional/integral/derivative (PID) controller and inserted in the the tubing. Details can be seen in Figure 4: the tip of the thermocouple measures the temperature after the heating jacket and just before the nozzle mounted on a full flow quick coupling. The superheated fluid is then sprayed through a hollow cone nozzle (**3**) into a chamber at a vacuum below 10 mbar.

**Figure 5:**
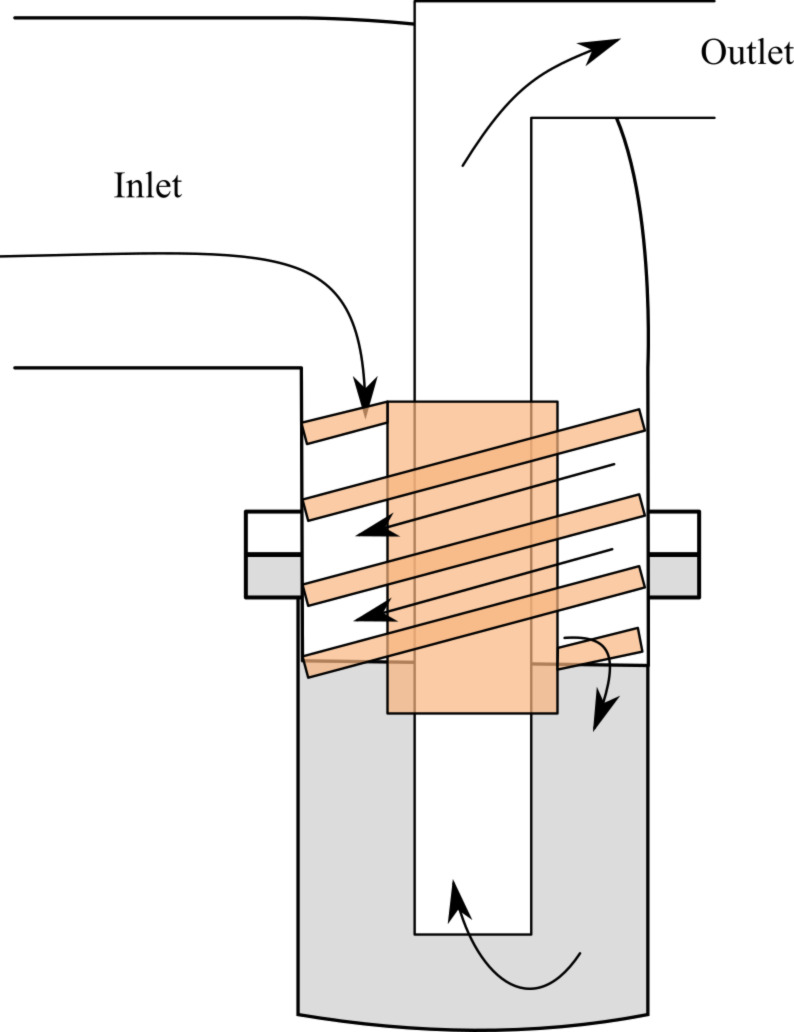
System for the product recovery: the cyclonic separator for vacuum (orange) and the interchangeable vessel (grey).

Figure 5 illustrates the recovery system of the solid particles from the gaseous flow coming from the evaporation. The cyclonic separators are made from the description of Chen and Tsai [115] who calculated a cut-off diameter of 21.7–49.8 nm. A glass flat flange reaction vessel allows one to gather the powder easily. One cyclone separates the aerosol, while the other unit can be isolated from the vacuum to recover the product. In this way the process operates continuously, spraying and separating the aerosol at any flow rate. At the end, the flow of gaseous solvent passes through a 35 m^3^/h vacuum pump; a condenser after the pump can recover the solvent for industrial installations. The standard operating conditions are: 40 bar of inlet pressure, 160 °C at the hollow cone nozzle, and an orifice diameter of 60 μm.

#### Versatility of SFE

The following parameters of SFE can be adapted, according to the solute and the desired particle size range:

- Type of solvent: The most suitable solvents for SFE are those having a low boiling point in the range of 30–70 °C. A high molar heat capacity helps to stabilize the fluid in its superheated state.
- Superheating temperature: Higher superheating temperature increases the evaporation rate, as mentioned earlier. The superheating temperature depends on the mass flow, the heat exchanger (surface, geometry and residence time) and the fluid properties.
- Pre-expansion pressure: The pre-expansion pressure has to be above the vapor pressure of the superheated solvent. That pressure should also be compatible with nozzle diameter and type, with for instance an operating range from 30 to 150 bar for sub-millimeter nozzles. The droplet size is also known to decrease at higher pressures. However a higher pressure involves a higher flow rate, and a degradation of the superheating.
- Saturation pressure and temperature: The vacuum pump specifications mainly dictate the saturation pressure and temperature of the spray cone in the atomization chamber. The volume and geometry of the chamber and the ones of the whole vacuum piping system have only a minor influence when reaching high pumping flow rate.
- Nozzle diameter: For most nozzles types like hollow cone nozzles, full cone nozzles, or flat jet nozzles, a reduced orifice diameter decreases the droplet size; it also increases the preexpansion pressure needed to guarantee a fine spraying.

Besides the innovative applications of energetic nanomaterials, such as the synthesis of ultrafine nanodiamonds [116–117] and reactivity enhancement [10], the versatility of SFE allows the crystallization at a sub-micrometer or nanometer scales of a wide range of organic compounds. In particular, cocrystals of medical materials are of interest for drug enhancement and were successfully processed through SFE at the nanoscale [118]. Inorganic nanoparticles were also produced on the nanoscale through SFE by Klaumuenzer et al. [119]. ZnO was crystallized from the precursor zinc acetate dihydrate dissolved in ethanol with addition of water. From early experiments, primary nanoparticles of 20 nm were found to be agglomerated in sub-size structures, whereas the slightly larger nanoparticles were found much less agglomerated using the classical wet method. However, the SFE clearly demonstrated the feasibility of faster and quite efficient crystallization of inorganic particles from precursors. Le Brize and Spitzer [120] processed energetic composite materials by SFE: a sub-micrometer structure was evidenced from SEM pictures and an higher degree of chemical interaction was also found from IR and Raman spectra. The high versatility of the SFE permits the processing of liquid (poly(ethylene glycol) (PEG) 400) and solid (PVP 40k) polymers to tune the RDX particle size distribution from the nanometer to the micrometer scale with controlled shapes [121]. The smallest particle diameter obtained was at 160 nm with a spherical morphology. Adding 0.05 wt % of PVP decreased the size of RDX by 34%, from around 500 nm to 320 nm, but also significantly improved the spheric shape. Additionally, all the synthesized RDX samples were less sensitive, especially toward electrostatic discharge.

## Conclusion

As displayed in Table 6, the smallest diameter of RDX is either obtained from wet techniques or from small-scale approaches which cannot be transferred to industry (PVD and laser ablation). Even if PVD has been successfully used in the semiconductor sector for our everyday electronic devices for decades, PVD applied on energetic materials will never be able to reach a production of several hundred of grams per hour. However, PVD is suitable for the current trend to create ”pyrotechnic integrated circuits”. Femtosecond laser ablation is used for nanoparticle synthesis of metal in solution at the laboratory scale. The colloids produced are found to be extremely stable. Used in dried conditions, a deposit of nanoparticles on a substrate could be obtained from a gas flow, or a dried powder could be collected within a cyclonic separator. This laser-based technique has been used to cut high energetic material quite safely [122] but nanoparticle production would be severely limited to high-added-value industrial applications due to low production rate and high operation cost. Besides those two aspects, neither methods would process advanced composites, with a binder for instance, or would be able to do concomitant or co-crystallization.

**Table 6:** Comparison and summary of the major techniques for the nanocrystallization of energetic materials with an industrial point of view.

Process	Working pressure(s)	Heating (°C)	Continuous	Scale-up	Limiting step(s)	Smallest size^a^

Sol–gel	atmospheric	no	no	−+	matrix, drying	100–150^b^
Antisolvent	atmospheric	70	could be	−	injection, drying	38
Milling	atmospheric	cooling	no	+	drying	160^c^
PVD	10^−4^ Pa^d^	100–200	no	−−	vacuum	50
Electrospray	atmospheric	no	could be	−	mass flow, electric field	400
ASES	12 MPa	yes and cooling	no	−−	scCO_2_^e^	micrometers
SEDS	35 MPa^d^	yes	no	−−	scCO_2_	micrometers
RESS	35 MPa → 0.1–5 MPa	yes and cooling	could be	−+	scCO_2_	200
RESS-AS	35 MPa → atmospheric	25	could be	−+	scCO_2_, drying	30^f^
Laser	atmospheric	no	no	−	mass flow	64
Ultrasonic	atmospheric	50–150	could be	−+	transducer	200–500
Spray drying	atmospheric	50–100	could be	++	evaporation ratio	400
SFE	5 MPa → 5 mbar	150	yes	++	vacuum	300

^a^Smallest pure RDX mean diameter reported in nm; ^b^XRD measurement; ^c^Freeze-dried from a 64 nm RDX slurry; ^d^Not available in the references, so the value is based on usual operating conditions; ^e^supercritical carbon dioxide; ^f^From DLS, no report about dried state.

The production of nanoparticles through wet techniques has become a common industrial chemical process. The European project, Sustainable Hydrothermal Manufacturing of Nanometerials (SHYMAN), aims to increase the production rate of a continuous hydrothermal process from 1–10 tons/year to 100 tons/year for inorganic nanomaterials [123]. Tsuzuki et al. [124] statistically studied which methods for inorganic nanosynthesis are mostly employed in industry: vapor (39% mainly chemical vapor deposition (CVD)) and liquid (45%) phase synthesis are the two main types of techniques. Since patents or brand marketing can restrain the choice of a technology, this distribution should not be interpreted as a way to estimate the robustness or versatility of the methods. Considering such wide adoption of wet techniques [125] and the knowledge from chemical engineering (homogenization in large reactor, processing of liquid flow, versatility, safety etc.), wet crystallization methods are a logical choice to process organic materials. However, unlike inorganic and metal nanoparticles, organic matter is very sensitive to drying as we previously saw for milling. Yet this delicate step is required since the reactivity of high energetic materials is fully exploited in the dried state. Freeze drying and supercritical drying seem to kinetically and partially prevent crystal growth from occurring. Only a complete growth inhibition will lead to the production of smaller nanoparticles under 100 nm from milling or antisolvent/cooling crystallization. From an industrial point of view, freeze or supercritical drying are batch-only processes. All current industrial drying process are not designed to tackle the fast growth of soft matter. Innovative techniques such as spin freezing [126] or spray drying enhance the processability, and potentially the performance, but the rigorous testing of their reliability is yet an open issue.

The ball milling techniques raise concerns about the purity of the product. It is well know that after such an extensive friction process, industrially milled ceramics cannot be used for high purity chemical processes [127]. Industry moved to other techniques such as vapor phase-based techniques to overcome that limitation in addition to others like the lake of control, local heating etc. Even with soft matter, similar issues can be expected; even small quantities of metallic impurities could catalyze the degradation of the explosive and/or sensitize it.

After 25 years of research and 10 years of process engineering, the supercritical fluid (SCF) technology has not convinced the industry and only marginal use for the specific commercial drug products have been reported [125]. First, the choice of the gas at industrial scales is returning to CO_2_ due to safety and affordability criteria. For instance, gases such as nitrous oxide or ethane have low critical values, but explosive mixtures can be generated. Trifluoromethane is inert, nonflammable and is usually a better solvent, but is significantly more expensive than CO_2_ and a potent greenhouse gas. Second, the main limitation, the solubility into scCO_2_, can be overcome by the addition of an organic cosolvent. Such a modification alters the environmentally safe nature of scCO_2_-based SCF and complicates the process by the need to remove any residual organic solvents. The aggregation phenomenon is commonly observed in SCF processes; further investigations on the role of different particle collection environments are needed, but RESS-AS processes greatly avoid the particle aggregation. The use of a liquid antisolvent with polymeric stabilizers has been found to be very effective. However, it compromises the recovery of a dry, pure powder, going back to square one with the drying issues previously discussed.

Spray techniques are commonly used in the industry, such as microencapsulation massively used for food [128–129], spray drying in pulmonary drug delivery for production of uniform and breathable size particles [130] or even thermal spray deposition of metallic material [131]. Spraying is a method which allows easy implementation of an installation and easy direct control over the injection. However, because of the low technological cost of atomizing nozzles and the low control over the spray itself, details and know-how are much more important than for other processes. Direct spray drying as a crystallization technique for RDX does not produce sub-micrometer-sized particles without the help of an additive and the SCF techniques are not suitable for industry. The need for an intermediate method in terms of pressure and temperature leads to the creation of the SFE technique, especially tailored for crystallization. SFE operates from 40–100 bar with an RDX solubility in acetone around 5 wt %, whereas scCO_2_ is formed from 74–500 bar for a solubility from null to 0.025 wt %.

The SFE technology has proven its high level of versatility and reliability, and is now entering advanced stages of development. Scale-up studies are performed and advanced in situ characterization is under investigation. Preliminary results using phase Doppler interferometry (PDA) reveals that SFE produces micrometer sized droplets with high velocities, typical features of interest for metastable crystalline structures.

For a deeper understanding of the crystallization of energetic materials at the nanoscale, a better comprehension of the resulting powder is needed. Beyond the issue of the reliability of the sensitivity values of energetic materials at the nanoscale [132] and tested at the laboratory scale [133], the pyrotechnic community should actively discuss the repeatability, the agglomeration of particles and the particle size distribution with accurate peak fittings.

## Supporting Information

File 1 CSV data on fine energetic materials.
